# AltitudeOmics: Resetting of Cerebrovascular CO_2_ Reactivity Following Acclimatization to High Altitude

**DOI:** 10.3389/fphys.2015.00394

**Published:** 2016-01-08

**Authors:** Jui-Lin Fan, Andrew W. Subudhi, James Duffin, Andrew T. Lovering, Robert C. Roach, Bengt Kayser

**Affiliations:** ^1^Centre for Translational Physiology, University of OtagoWellington, New Zealand; ^2^Department of Surgery and Anaesthesia, University of OtagoWellington, New Zealand; ^3^Department of Emergency Medicine, Altitude Research Center, University of Colorado DenverAurora, CO, USA; ^4^Department of Biology, University of Colorado Colorado SpringsColorado Springs, CO, USA; ^5^Department of Physiology, University of TorontoToronto, ON, Canada; ^6^Department of Anaesthesiology, University of TorontoToronto, ON, Canada; ^7^University Health NetworkToronto, ON, Canada; ^8^Department of Human Physiology, University of OregonEugene, Oregon, OR, USA; ^9^Institute of Sports Sciences, Faculty of Biology and Medicine, University of LausanneLausanne, Switzerland; ^10^Department of Physiology, Faculty of Biology and Medicine, University of LausanneLausanne, Switzerland

**Keywords:** cerebral blood flow, cerebral blood flow regulation, cerebral hemodynamics, high altitude, transcranial Doppler

## Abstract

Previous studies reported enhanced cerebrovascular CO_2_ reactivity upon ascent to high altitude using linear models. However, there is evidence that this response may be sigmoidal in nature. Moreover, it was speculated that these changes at high altitude are mediated by alterations in acid-base buffering. Accordingly, we reanalyzed previously published data to assess middle cerebral blood flow velocity (MCAv) responses to modified rebreathing at sea level (SL), upon ascent (ALT1) and following 16 days of acclimatization (ALT16) to 5260 m in 21 lowlanders. Using sigmoid curve fitting of the MCAv responses to CO_2_, we found the amplitude (95 vs. 129%, SL vs. ALT1, 95% confidence intervals (CI) [77, 112], [111, 145], respectively, *P* = 0.024) and the slope of the sigmoid response (4.5 vs. 7.5%/mmHg, SL vs. ALT1, 95% CIs [3.1, 5.9], [6.0, 9.0], respectively, *P* = 0.026) to be enhanced at ALT1, which persisted with acclimatization at ALT16 (amplitude: 177, 95% CI [139, 215], *P* < 0.001; slope: 10.3%/mmHg, 95% CI [8.2, 12.5], *P* = 0.003) compared to SL. Meanwhile, the sigmoidal response midpoint was unchanged at ALT1 (SL: 36.5 mmHg; ALT1: 35.4 mmHg, 95% CIs [34.0, 39.0], [33.1, 37.7], respectively, *P* = 0.982), while it was reduced by ~7 mmHg at ALT16 (28.6 mmHg, 95% CI [26.4, 30.8], *P* = 0.001 vs. SL), indicating leftward shift of the cerebrovascular CO_2_ response to a lower arterial partial pressure of CO_2_ (PaCO_2_) following acclimatization to altitude. Sigmoid fitting revealed a leftward shift in the midpoint of the cerebrovascular response curve which could not be observed with linear fitting. These findings demonstrate that there is resetting of the cerebrovascular CO_2_ reactivity operating point to a lower PaCO_2_ following acclimatization to high altitude. This cerebrovascular resetting is likely the result of an altered acid-base buffer status resulting from prolonged exposure to the severe hypocapnia associated with ventilatory acclimatization to high altitude.

## Introduction

During metabolic and respiratory-mediated pH perturbations, both cerebral blood flow (CBF) and ventilation can be expressed as a single linear function of the hydrogen ion concentration ([H^+^] in extracellular fluid, and therefore cerebrospinal fluid (CSF) (Mitchell et al., 1965; Pappenheimer et al., 1965; Fencl et al., 1966, 1969; Skinhoj, 1966; Betz and Heuser, 1967; Severinghaus and Lassen, 1967; Lassen, 1968; Kontos et al., 1977; Warner et al., 1987; Smith et al., 1988; Toda et al., 1989). Since bicarbonate (${\text{HCO}}_{3}^{-}$) is the main buffer base in the CSF compartment (Siesjö, 1972), changes in partial pressure of arterial carbon dioxide (PaCO_2_) will lead to corresponding changes in [H^+^] unless compensatory adjustment in [${\text{HCO}}_{3}^{-}$] occurs (Mitchell et al., 1965). Accordingly, CSF [${\text{HCO}}_{3}^{-}$] is an important determinant of cerebrovascular and ventilatory responses during respiratory acid-base disturbances.

During prolonged exposure to hypoxia, the hyperventilation-induced hypocapnia and associated respiratory alkalosis lead to a compensatory renal ${\text{HCO}}_{3}^{-}$ excretion, thereby lowering [${\text{HCO}}_{3}^{-}$] and returning pH toward homeostatic values in the arterial and CSF compartment (Dempsey et al., 1974, 1975, 1978; Forster et al., 1975). At sea-level, reducing CSF [${\text{HCO}}_{3}^{-}$] with oral ammonium chloride administration increases the cerebrovascular and ventilatory responsiveness to CO_2_, and elicits a resetting of these responses to lower PaCO_2_ values (Fencl et al., 1969). This reduction in CSF [${\text{HCO}}_{3}^{-}$] and associated base buffering change leads to a greater increase in [H^+^] for a given rise in PaCO_2_ (Siesjö, 1972). Such changes in acid-base buffering in the CSF compartment accounts for the enhanced CBF and ventilatory responsiveness to CO_2_ observed at high altitude (Mathew et al., 1983; Schoene et al., 1990; Fan et al., 2014), along with the leftward shift in the ventilatory CO_2_ sensitivity (Crawford and Severinghaus, 1978; Mathew et al., 1983; Fan et al., 2012, 2014). Pioneering work by Severinghaus (1965) estimated that CBF would be 78% above pre-altitude values if arterial partial pressures of O_2_ (PaO_2_) and CO_2_ were returned to sea-level values following 3–5 days at 3800 m. Similarly, data from Fan et al. (2010, 2012) indicated a leftward shift in the CBF response to CO_2_ following prolonged high altitude exposure. Since there is a delayed onset of compensatory metabolic acidification we would expect to observe no such resetting of the cerebrovascular CO_2_ reactivity during acute high altitude exposure. However, no studies have identified the magnitude of this cerebrovascular resetting during acute and chronic exposure to high altitude.

Under constant arterial blood pressure, the overall relationship of PaCO_2_ and CBF is sigmoidal with a linear portion between PaCO_2_ of 25–65 mmHg (Madden, 1993). While the majority of the literature is focused on the linear portion of this response, sigmoid fitting might be more appropriate for the assessment of cerebrovascular CO_2_ reactivity at high altitude due to the severe hypocapnia resulting from ventilatory acclimatization to high altitude. Further, the use of a linear model may miss a number of physiological parameters (e.g., the optimal operating point) and physical constraints of the cerebral vasculature (e.g. vessels' finite dilation/constriction and associated vascular reserves; Battisti-Charbonney et al., 2011). Accordingly, sigmoid curve fitting could provide additional insight to the changes in cerebrovascular CO_2_ reactivity at high altitude (such as changes in vascular dilation or constriction reserves and a shifting of the operating point).

Using the sigmoid curve fitting method described by Battisti-Charbonney et al. (2011), we reanalyzed our cerebrovascular CO_2_ reactivity data from rebreathing tests as previously reported in Fan et al. (2014). We tested the hypotheses that: (1) ascent to high altitude is accompanied by a leftward shift as well as an increase in the cerebrovascular response to CO_2_ upon ascent to 5260 m and following acclimatization; (2) these changes in cerebrovascular CO_2_ reactivity would be abolished once we account for the acid-base change by plotting CBF against [H^+^] instead of PCO_2_; and (3) the use of sigmoid fitting will reveal changes in vascular reserve at high altitude.

## Materials and methods

### Subject recruitment and screening

This study was conducted as part of the AltitudeOmics project. Following institutional ethics approval, young (19–23 years old), healthy, sea level residents were recruited from the greater Eugene, Oregon area (elevation 130 m). Potential subjects were screened to exclude anyone who was born or had lived at altitudes >1500 m for more than 1 year or had traveled to altitudes >1000 m in the past 3 months. After obtaining written consent, physical exams and the Army Physical Fitness Test (push-ups, sit-ups, and a 3.2-km run) were performed to verify health and fitness status (Subudhi et al., 2014b).

### Ethical approval

The study was performed according to the *Declaration of Helsinki* and was approved by the Institutional Review Boards of the Universities of Colorado and Oregon and by the Human Research Protection Office of the U.S. Department of Defense. All participants were informed regarding the procedures of this study, and written informed consent was given prior to participation.

### Experimental design

After familiarization with the experimental procedures outlined below (visit one), twenty-one subjects underwent three experimental trials: near sea level (SL: 130 m, barometric pressure: 749 mmHg) and on day 1 and 16 following ascent to 5260 m (ALT1 and ALT16). Approximately 4 weeks following SL measurements in Eugene, Oregon, the subjects were flown to La Paz, Bolivia. They spent two nights at low altitude (1525 m in Coroico, Bolivia) before being driven to the Chacaltaya Research Station at 5260 m, while breathing supplemental oxygen (ALT1). Subjects acclimatized to altitudes ranging from 3800 to 5260 m over the next 15 days, with most of the time (75%) spent at 5260 m. On the 16th day (ALT16), measurements were repeated (Subudhi et al., 2014b).

### Experimental protocol

A 22-gauge catheter was inserted into a radial artery at least 1 h prior to instrumentation. Subjects were seated in an upright position for 15 min, while sensors were placed to measure physiological variables of interest (Subudhi et al., 2014b). Following 10–15 min of quiet rest in a seated position, each experimental testing session comprised of: (a) instrumentation; (b) 10 min room air baseline; (c) 10 min steady-state with partial pressure of end-tidal CO_2_ (PETCO_2_) and O_2_ (PETO_2_) clamped at 40 mmHg and 300 mmHg, respectively, and (d) assessment of cerebrovascular CO_2_ reactivity with a modified rebreathing test. For each subject, all ALT measurements were carried out around the same time of day to minimize any confounding effect by circadian rhythm. Measurements were taken upon arrival at ALT1 to minimize the influence of acute mountain sickness (AMS). Likewise, no symptoms of AMS were observed at ALT16.

Throughout the protocol, the subjects sat upright and breathed through a mouthpiece attached to a two-way non-rebreathing valve (Hans-Rudolph 2700, Hans-Rudolph Inc., Shawnee, KS, USA). The breathing circuit allowed switching from room air to either an end-tidal clamping system or a rebreathing system.

### Modified rebreathing method

The rebreathing bag was filled with gas to achieve inspired PCO_2_ and PO_2_ of 0 and 300 mmHg, respectively, upon rebreathing at each altitude. Hyperoxia was used to abolish the potentially confounding influence of changes in PaO_2_ at SL, ALT1, and ALT16 on our cerebrovascular CO_2_ reactivity estimates. Subjects were instructed to hyperventilate for 3 min to lower and then maintain PETCO_2_ at ~18 mmHg at both sea level and 5260 m (in background PETO_2_ > 250 mmHg). Subjects were then switched to the rebreathing bag, and following two initial deep breaths to mix the gas from the bag with that in the respiratory system, they were instructed to breathe *ad libitum*. The rebreathing tests were terminated when PETCO_2_ reached 50 mmHg, PETO_2_ dropped below 200 mmHg or the subject reached the end of his/her tolerance.

## Measurements

### Cerebral blood flow velocity

Middle cerebral artery velocity (MCAv, an index of cerebral blood flow) was measured in the left middle cerebral artery using a 2-MHz pulsed Doppler ultrasound system (ST3, Spencer technology, Seattle, WA, USA). The Doppler ultrasound probe was positioned over the left temporal window and held in place with an adjustable plastic headband (Marc 600 Headframe, Spencer technology, Seattle, WA, USA). The signal was acquired at depths ranging from 43 to 54 mm. Signal quality was optimized and an M-mode screen shot was recorded to facilitate subsequent probe placements.

### Arterial blood pressure

Beat-to-beat arterial blood pressure (ABP) was measured from an arterial catheter inserted in a radial artery, and connected to a calibrated, fluid-filled, disposable pressure transducer positioned at the level of the heart (DELTRAN II, Utah Medical, Salt Lake City, UT, USA).

### End-tidal gases

PETCO_2_ and PETO_2_ were measured using fast responding gas analyzers (O_2_Cap Oxygen analyzer, Oxigraf, Mountain View, CA, USA). The gas analyzers were calibrated using gas mixtures of known concentrations of O_2_ and CO_2_ prior to each testing session.

### Arterial blood gases

**Figure 1:**
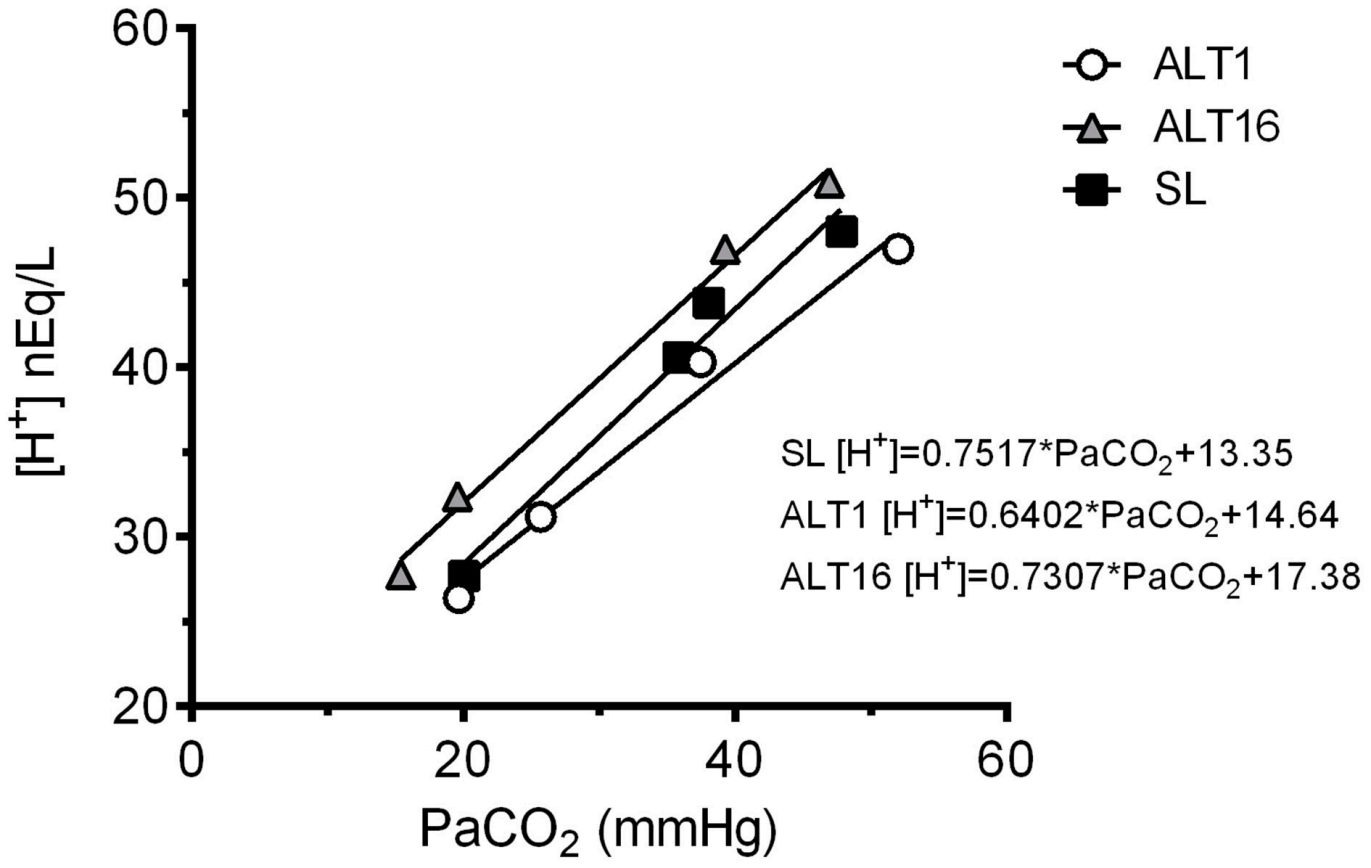
**Representative example of [H^+^] plotted against PaCO_2_ at sea level (SL), upon ascent (ALT1), and following 16 days acclimatization to 5260 m (ALT16) for one subject**.

For each subject, arterial blood samples were taken four times at SL, ALT1 and ALT16 (room air breathing, PETCO_2_ clamped at 40 mmHg, hyperventilation and end of rebreathing). Hydrogen ion concentration ([H^+^]) was calculated from the arterial pH values using the Siggaard-Andersen equation (OSA.exe, freely available at http://www.siggaard-andersen.dk). The [H^+^] values were plotted against PaCO_2_ (Figure 1) and the relationship between these two variables was obtained using linear regression (Prism 6.0b, GraphPad Software Inc., San Diego, CA, USA). The equation of this [H^+^]-PaCO_22_ slope was subsequently used to calculate the corresponding [H^+^] values for the range and the midpoint of the sigmoid curves for each rebreathing maneuver (Figure 1).

### Data acquisition

All analog data were sampled and recorded at 200 Hz on a PC for off-line analysis (ADInstruments Powerlab 16/30, Dunedin, New Zealand).

## Data analysis

### Rebreathing analysis (Figure 2)

As first detailed by Battisti-Charbonney et al. (2011), the following assumptions were made regarding the analysis of the rebreathing data: (i) hyperventilation lowers PETCO_2_ sufficiently to produce full hypocapnia-induced vasoconstriction; (ii) as PETCO_2_ increases from a hypocapnic value during hyperventilation to increasingly hypercapnic values during rebreathing, full vasodilation is achieved; the shape of this relationship is assumed to be sigmoidal with a linear portion in the middle of the curve (Claassen et al., 2007); and (iii) as hypercapnia increases beyond the point of maximal CO_2_-induced vasodilation, CBF becomes directly dependent on perfusion pressure. Based on these assumptions, cerebrovascular CO_2_ reactivity can be summarized as the vasodilatory and vasoconstrictive response of the cerebral blood vessels. The “midpoint” of the sigmoid curve then represents the optimal point of the vessels' capacity to dilate and constrict (i.e., the optimal operating point), while the upper and lower plateaus of the sigmoid response represent the physical constraints of the cerebral vascular reserve. Meanwhile, the range of the sigmoid curve reflects the PCO_2_ range of the linear portion of the sigmoid curve, and the amplitude represents the overall extent of this response.

The rebreathing data were first reduced to 1-s averages across the entire rebreathing period. MCAv values were then converted to a percentage change from MCAv values observed at resting PETCO_2_. Since the ABP-CO_2_ response is enhanced at high altitude (Ainslie and Burgess, 2008; Fan et al., 2014; Willie et al., 2015) and known to potentially confound estimates of cerebrovascular CO_2_ reactivity (Betz, 1968; Regan et al., 2014), a PETCO_2_ threshold (T_ABP_) was first identified as the point from which mean ABP rose more than 0.5 mmHg per mmHg change in PETCO_2_. This threshold was identified by fitting the mean ABP response to PETCO_2_, with two straight lines above and below the T_ABP_ using linear regression, and restricting the slope of the lower portion to < 0.5 mmHg change in ABP per mmHg change in PETCO_2_. The MCAv response to PETCO_2_ was then divided into two portions below and above the T_ABP_ and the portion of MCAv-PETCO_2_ response below T_ABP_ was fitted with a sigmoid curve as described by Battisti-Charbonney et al. (2011), thereby minimizing the confounding influence of ABP on the MCAv-CO_2_ response. To minimize the sum of squares for non-linear regression (Levenberg-Marquardt algorithm) we used the equation:

$$\begin{array}{l} \text{MCAv}=minimum+(amplitude/(1+\text{exp}(-({\text{PETCO}}_{2}-\\ \;midpoint)/range))) \end{array}$$

**Figure 2:**
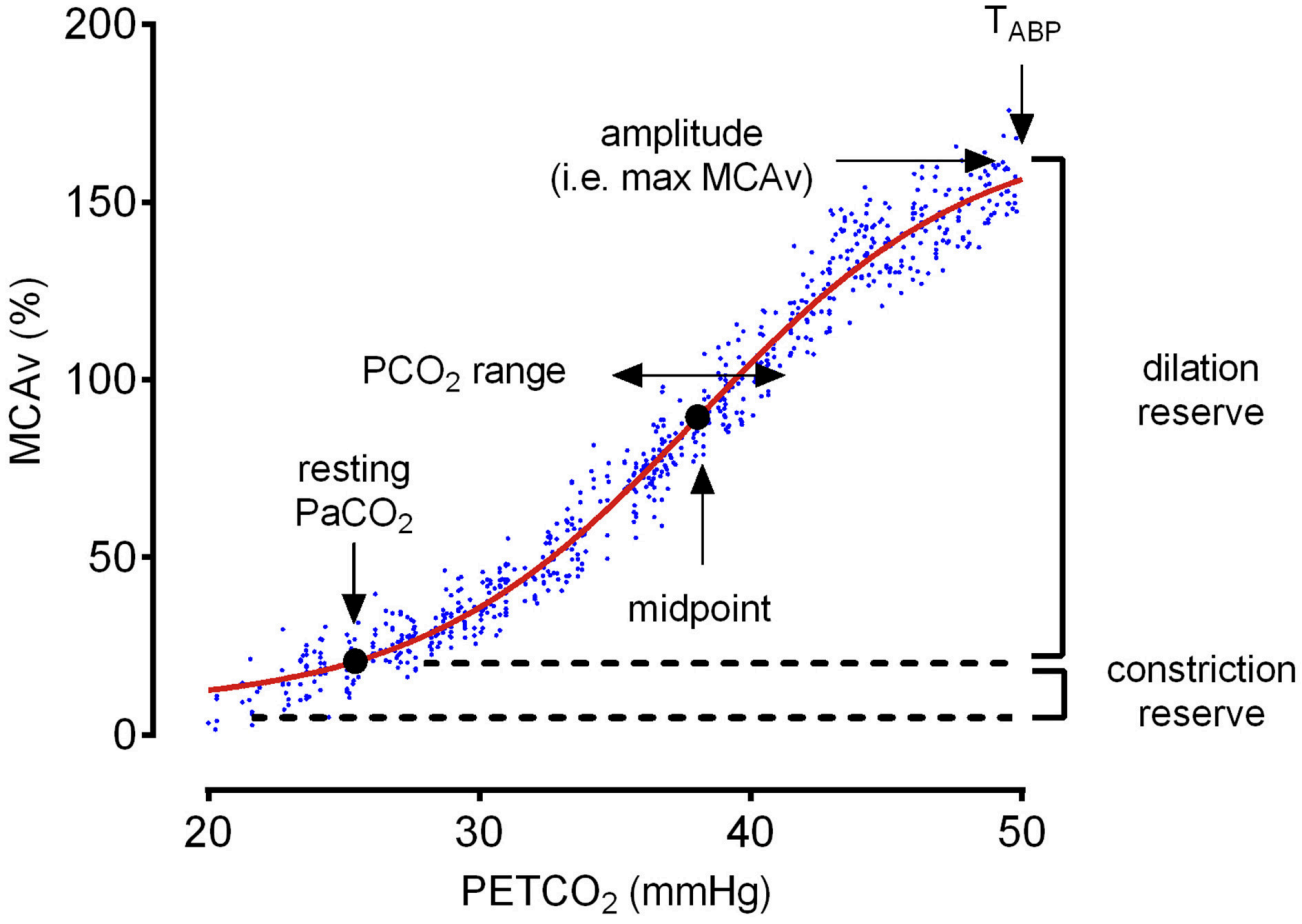
**A representative example of an individual rebreathing test and sigmoid curve fitting upon ascent to 5260 m (ALT1)**.

Where MCAv is the dependent variable in %, PETCO_2_ is the independent variable in mmHg, *minimum* is the minimum MCAv determined from the mean MCAv of the hypocapnic (hyperventilation) region, *amplitude* is the amplitude of the response (i.e., the maximum MCAv value), *midpoint* is the PCO_2_ value at the center of the sigmoid curve, and *range* is the PCO_2_ range of the linear portion of the sigmoid (an inverse reflection of the slope of the linear portion). The slope of the sigmoid curve (i.e., cerebrovascular CO_2_ reactivity) is subsequently calculated from the linear portion of the response. The vasoconstriction reserve was calculated as the difference between minimum MCAv and MCAv at resting PaCO_2_, while the vasodilation reserve was calculated as the difference between the MCAv at T_ABP_ (or amplitude if no T_ABP_ was observed during rebreathing) and MCAv at resting PaCO_2_, expressed at percentage MCAv (Figure 2).

The alignment and fitting processes were performed using custom written graphic analysis routines (LabVIEW, Nation Instruments, Austin, TX, USA). The fitting program provided an r-square of the sigmoid and linear fits.

### Statistical analysis

Due to logistical impacts on planning and transportation, not all subjects were able to participate in all high-altitude studies; please see the Figures and Table for complete sample size reporting for each procedure. After calculating descriptive statistics [mean, 95% confidence interval (CI)], mixed model linear regression analysis (diagonal repeated covariance assumed) (IBM® SPSS® Statistics version 21, IBM® Corporation, Armonk, NY, USA) was used to evaluate the effects of altitude exposure and acclimatization (between SL, ALT1 and ALT16) on the midpoint, amplitude and range of the fitted sigmoid curves. Post hoc tests were performed on the midpoint, amplitude and range of the sigmoid curves between SL, ALT1 and ALT16 using the Holm-Sidak adjustment for multiple comparisons (α = 0.05) (IBM® SPSS® Statistics version 21, IBM® Corporation, Armonk, NY, USA). Cohen's *d*, as a measure of effect size, was calculated as *d* = (*M*_1_ – *M*_2_)/*SD*, where *M*_1_ and *M*_2_ are means of group 1 and 2; *SD* is standard deviations of pooled data. The effect size was considered negligible when *d* < 0.2, small when *d* ≥ 0.2, moderate when *d* ≥ 0.5, large when *d* ≥ 0.8, and very large when *d* ≥ 1.3 (Sullivan and Feinn, 2012). Results were considered significant at the alpha level < 0.05. A priori power calculations (α = 0.05, β = 0.20) were used to determine sample size and limit Type II error.

## Results

Detailed baseline characteristics of the 21 (nine women; age 21 ± 1 years) subjects participating in AltitudeOmics were presented elsewhere (Subudhi et al., 2014a). All 21 subjects performed the modified rebreathing test at SL and ALT16. However, 4 of the 21 subjects did not perform the modified rebreathing at ALT1. After excluding data due to unstable ABP during rebreathing, 13, 9, and 7 trials met the inclusion criteria at SL, ALT1, and ALT16, respectively, and were included in the analysis. Sigmoid curve fitting was applied to the remaining trials with *R*^2^ of 0.89 ± 0.05 (mean ± *SD*).

### Cerebrovascular CO_2_ reactivity

**Figure 3:**
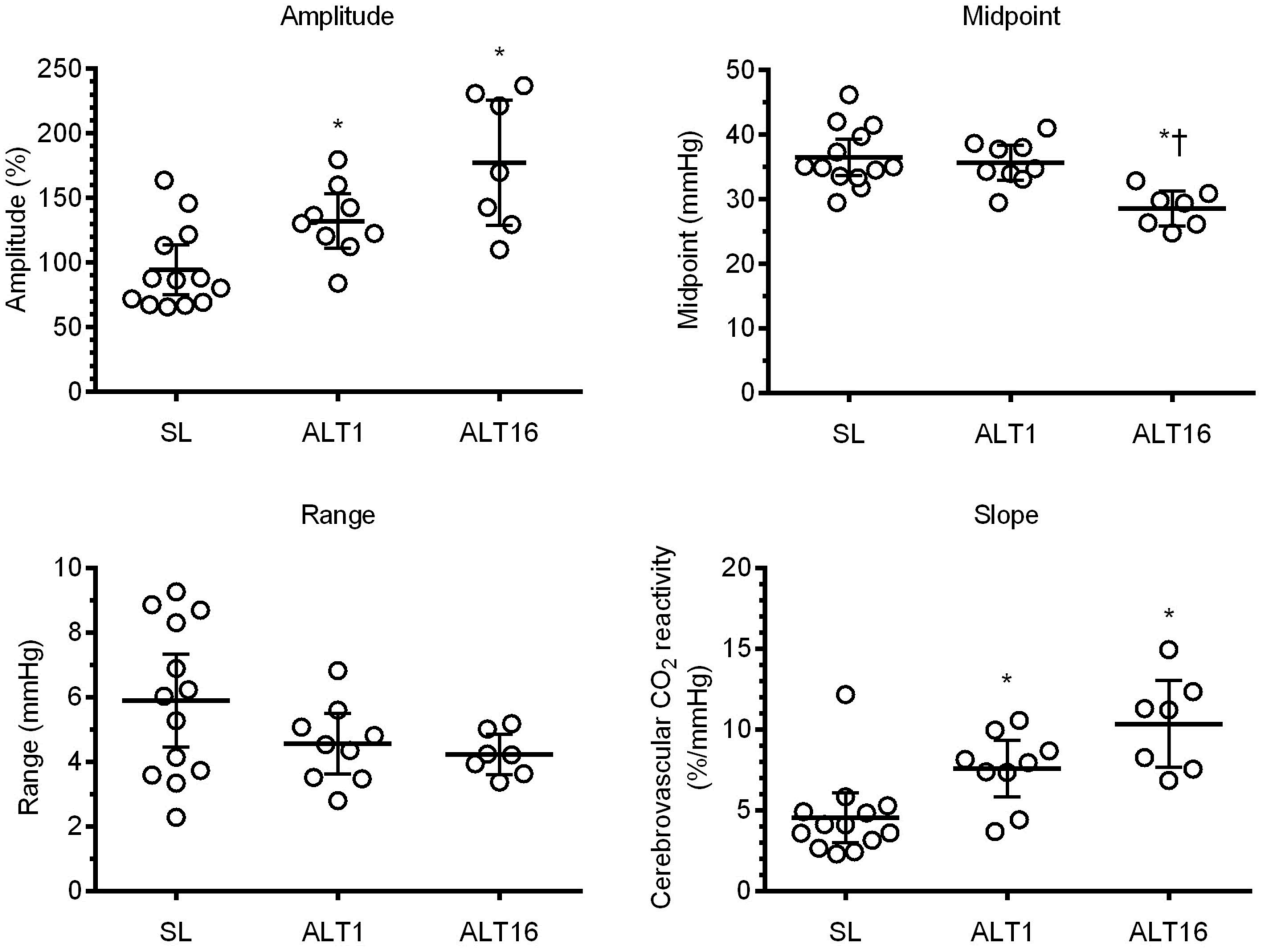
**Modified rebreathing parameters at sea level (SL), upon ascent (ALT1), and following 16 days acclimatization to 5260 m (ALT16)**. Data expressed as mean ± 95% confidence interval. ^*^ different from SL (*P* < 0.05); † different from ALT1 (*P* < 0.05).

**Figure 4:**
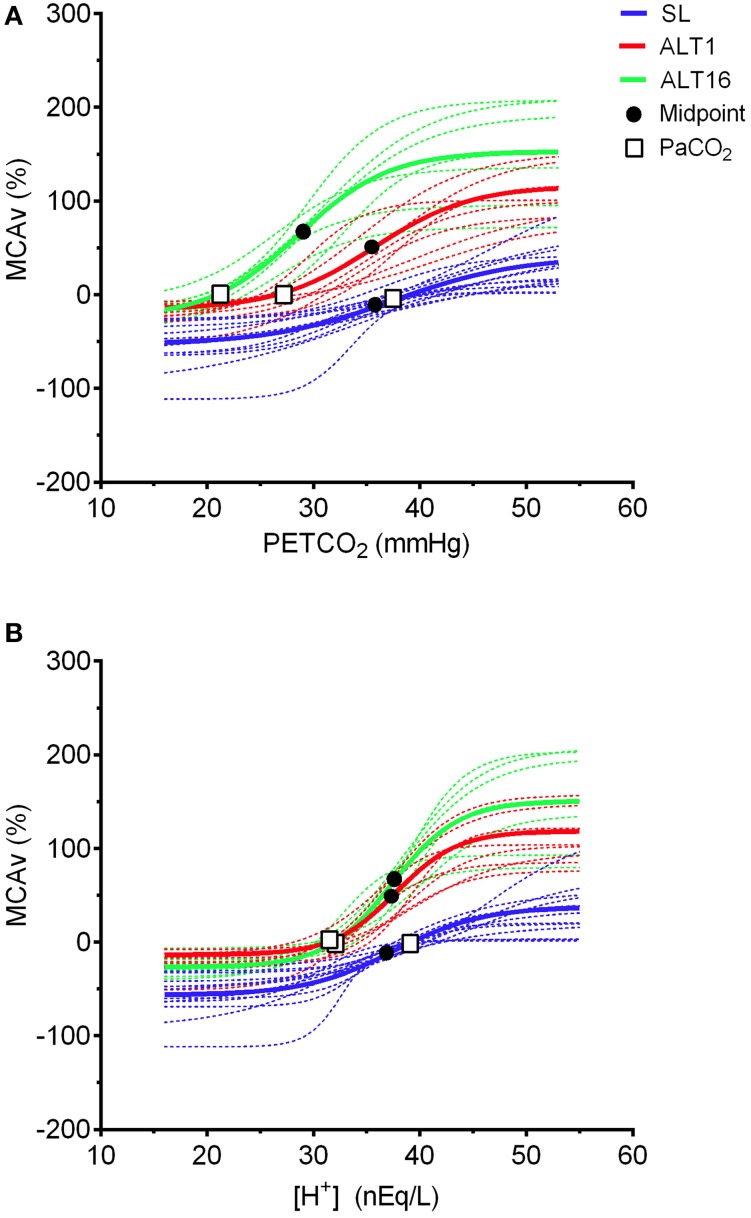
**Fitting of the individual (dotted line) and group (solid line) MCAv response to PETCO_2_ (A) and [H^+^] (B) during modified rebreathing at sea level (SL), upon ascent (ALT1), and following 16 days acclimatization to 5260 m (ALT16)**. Our data indicates that when MCAv is plotted against [H^+^] rather than PETCO_2_, the leftward shift in the sigmoid curve at ALT16 is abolished. Moreover, **(B)** demonstrates that the midpoints of the sigmoid curves are around 37 nEq/L regardless of the altitude or acclimatization status.

No difference was observed in the PETCO_2_ range of the linear portion of the sigmoid between SL and ALT1 (*d* = 1.0, *P* = 0.149). Likewise, the PETCO_2_ range was unaltered following acclimatization at ALT16 when compared to SL (*d* = 1.2, *P* = 0.060) or to ALT1 (*d* = 0.3, *P* = 0.149 vs. ALT1, Table 1). Upon ascent to 5260 m (ALT1), the amplitude of the MCAv response was increased by ~34% (*d* = 5.8, *P* = 0.024 vs. SL), and further increased by ~82% at ALT16 (*d* = 11.1, *P* < 0.001 vs. SL, Table 1). However, the amplitude of the sigmoid MCAv response was not different between ALT1 and ALT16 (*d* = 7.3, *P* = 0.101, Table 1). As a result of these increases in amplitude of the MCAv response, the linear portion of the sigmoid curve was enhanced by ~67% at ALT1 (*d* = 1.8, *P* = 0.026) and by ~129% at ALT16 (*d* = 3.0, *P* = 0.003) compared to SL, while no difference was observed from ALT1 to ALT16 (*d* = 1.7, *P* = 0.175, Table 1). The PETCO_2_ midpoint of the sigmoid curve was unchanged upon ascent to 5260 m (*d* = 0.5, *P* = 0.982 vs. SL), while it was reduced by ~8 mmHg (i.e., shifted leftward) following acclimatization at ALT16 (*d* = 3.3, *P* = 0.001 vs. SL, Table 1). Accordingly, the PETCO_2_ midpoint was lower at ALT16 by ~7 mmHg compared to initial exposure to high altitude (*d* = 3.1, *P* = 0.005 vs. ALT1, Table 1). These findings indicate that cerebrovascular CO_2_ reactivity was enhanced upon ascent to 5260 m, resulting in both upward shifting and steeping of the sigmoid curve compared to sea level (Figure 3). Following acclimatization, at ALT16, there was a leftward shift of the sigmoid curve (Figure 4A), while, cerebrovascular CO_2_ reactivity did not differ greatly between ALT1 and ALT16 (Figure 3).

**Table 1:** **Sigmoid curve fitting parameters during modified rebreathing**.

**Variable**	**SL**		**ALT1**		**ALT16**	
***n***	**13**		**9**		**7**	
	**Mean**	**95% CI**	**Mean**	**95% CI**	**Mean**	**95% CI**
Amplitude (%)	95	[77–112]	129^*^	[111–147]	177^*^	[139–216]
Midpoint (mmHg)	36.5	[34.0–39.0]	35.4	[33.1–37.7]	28.6^*^^†^	[26.4–30.8]
Range (mmHg)	5.9	[4.6–7.2]	4.5	[3.7–5.3]	4.2	[3.7–4.7]
Cerebrovascular CO_2_ reactivity (%/mmHg)	4.5	[3.1–5.9]	7.5^*^	[6.0–9.0]	10.3^*^	[8.2–12.5]
Constriction reserve (%)	56	[42–70]	25^*^	[16–34]	25^*^	[15–36]
Dilation reserve (%)	20	[7–34]	103^*^	[82–125]	112^*^	[84–139]
**PLOTTED AGAINST [H^+^]**						
Midpoint (nEq/L)	37.5	[35.0–40.1]	37.7	[36.7–38.7]	37.4	[35.3–39.4]
Range (nEq/L)	4.2	[3.3–5.1]	2.9	[2.4–3.4]	3.1	[2.6–3.6]
Cerebrovascular CO_2_ reactivity (%/nEq/L)	6.4	[4.5–8.4]	11.4^*^	[9.3–13.5]	14.1^*^	[11.2–17.1]

Different from SL(

* ) and ALT1 (

† *), P < 0.05*.

Compared to SL, the vasoconstriction reserve was lowered by ~31% at both ALT1 (*d* = 1.2, *P* = 0.008) and at ALT16 (*d* = 1.2, *P* = 0.012), while the vasodilation reserve was increased by ~83% at ALT1 (*d* = 1.67, *P* < 0.001) and by ~92% at ALT16 (*d* = 1.7, *P* < 0.001, Table 1). No differences were observed between ALT1 and ALT16 for the constriction (*d* = 0.0, *P* = 1.000) and dilation reserves (*d* = 0.2, *P* = 0.945, Table 1).

### Acid-base buffering

When expressing MCAv against [H^+^], the range was unaltered following ascent at ALT1 (*d* = 1.0, *P* = 0.137 vs. SL) or with acclimatization at ALT16 (*d* = 0.9, *P* = 0.171), and no difference in the [H^+^] range of the linear sigmoid portion was observed between ALT1 and ALT16 (*d* = 0.2, *P* = 0.993, Table 1). Similarly, the midpoint of the sigmoid curve occurred at similar [H^+^] at SL, ALT1 (*d* = 0.0, *P* = 1.000 vs. SL) and ALT16 (*d* = 0.1, *P* = 0.999, Table 1). Meanwhile, cerebrovascular CO_2_ reactivity was enhanced upon ascent to ALT1 by ~78% (*d* = 2.4, *P* = 0.012 vs. SL) and remained elevated at ALT16 (*d* = 3.4, *P* = 0.004, Table 1). No difference was found in cerebrovascular CO_2_ reactivity between ALT1 and ALT16 (*d* = 1.4, *P* = 0.396, Table 1).

## Discussion

In the present study, we examined the changes in cerebrovascular parameters during modified rebreathing using sigmoid curve fitting analysis. Using sigmoid fitting, we found: (1) the increase in cerebrovascular CO_2_ reactivity at ALT1 is mediated by an enhanced sensitivity of the cerebral blood vessels to H^+^ (Figure 4B); (2) there is a leftward shift of MCAv-CO_2_ response with acclimatization at ALT16 (Figure 4A), indicating a resetting of the cerebrovascular CO_2_ reactivity sigmoid midpoint to a lower PaCO_2_ with chronic hypoxia; (3) this leftward shift in cerebrovascular CO_2_ reactivity is abolished once we account for the changed acid-base buffering status; and 4) despite this cerebrovascular resetting, the vascular constriction reserve is reduced at high altitude. From our findings, it appears that this cerebrovascular resetting to a lower range of PaCO_2_, mediated by changes in acid-base buffering, is likely a serendipitous consequence of acclimatization to high altitude. Importantly, this resetting restores cerebrovascular control during the persistent severe hypocapnia associated with prolonged exposure to high altitude.

CBF regulation during both acute and chronic hypoxia can be summarized as the *net effect* of hypoxia-induced vasodilation and hypocapnia-induced vasoconstriction of the cerebral blood vessels (Xu and Lamanna, 2006; Brugniaux et al., 2007). During initial exposure to severe hypoxia, the vasodilatory drive in the cerebral blood vessels typically exceeds that of the constrictor drive, thereby elevating CBF (Severinghaus et al., 1966; Subudhi et al., 2014c). During acclimatization, the progressive increase in ventilatory drive lowers PaCO_2_ and elevates PaO_2_ (Brugniaux et al., 2007), resulting in greater vasoconstrictor drive and restoring of CBF to pre-exposure values (Severinghaus et al., 1966; Subudhi et al., 2014c). In the absence of a cerebrovascular resetting, the progressive increase in ventilatory drive and associated hyperventilation-induced hypocapnia with acclimatization would eventually lead to cerebral hypoperfusion. Therefore, the observed resetting of cerebrovascular CO_2_ reactivity to a lower PCO_2_ operating point mitigates the adverse effects of hypocapnia-induced vasoconstriction, thus enabling the cerebral vessels to better regulate CBF during prolonged high altitude exposure.

### Altered acid-base buffering

The CSF is a bicarbonate-containing fluid which acts as an important acid-base buffer in the regulation in CSF pH (Siesjö, 1972). Since there are negligible concentrations of protein and other non-bicarbonate buffers in the CSF (Davson, 1967), changes in [${\text{HCO}}_{3}^{-}$] reflect changes in buffer base (Siesjö, 1972). As elegantly summarized by Fencl et al. (1969), the regulation of CBF and ventilation are components of the same homeostatic system, where the input consists of [H^+^] in the CSF compartment, which is determined by [${\text{HCO}}_{3}^{-}$] and PCO_2_. In this system, the respiratory loop regulates PaCO_2_ (and therefore [H^+^] controlling CBF), while the cerebrovascular loop regulates the PCO_2_ gradient between arterial and CSF compartment (and therefore [H^+^] at the level of the central chemoreceptors). Importantly, the bicarbonate concentration provides the link between the two regulatory loops of this system. Under conditions of metabolic acidosis or alkalosis, CSF [${\text{HCO}}_{3}^{-}$] is regulated to reduce CSF pH variations to one tenth of that occurring in the arterial compartment (Fencl et al., 1969; Dempsey et al., 1978). Since a CSF pH change of 0.2 unit covers the entire ventilatory response from life-threatening hypoventilation to maximal hyperventilation (Pappenheimer, 1967), a close regulation of CSF pH is critical for normal respiratory function. As CSF [${\text{HCO}}_{3}^{-}$] is an important link between cerebrovascular and ventilatory control, any bicarbonate-mediated changes in ventilatory CO_2_ sensitivity should be reflected in cerebrovascular CO_2_ reactivity. Moreover, changes in CSF [${\text{HCO}}_{3}^{-}$] would lead to proportional changes in the PCO_2_ range within which CBF and ventilation are regulated. In support, we found there was a leftward shift of the PaCO_2_-[H^+^] relationship at ALT16 (Table 1).

#### Enhanced sensitivity to H^+^ during acute exposure

During respiratory alkalosis, as observed during hypoxic exposure, a reduced CSF [${\text{HCO}}_{3}^{-}$] leads to a greater increase in [H^+^] for a given rise in PaCO_2_ (Siesjö, 1972). Previously, we speculated these changes in acid-base buffering following prolonged hypoxic exposure could account for the enhanced cerebrovascular CO_2_ reactivity during initial exposure to high altitude (Fan et al., 2010). Accordingly, any increase in cerebrovascular CO_2_ reactivity at high altitude should be abolished when plotting MCAv changes against [H^+^] instead of PCO_2_. However, in the present analysis we found the increase in sigmoid slope at ALT1 persisted when we plotted MCAv against [H^+^] (Figure 4B), which indicates that this increase in cerebrovascular CO_2_ reactivity upon ascent to high altitude is mediated by an enhanced H^+^ sensitivity in the cerebral vasculature *per se*, rather than altered acid-base balance only.

The presence of a cerebrovascular function resetting at high altitude was first alluded to by Severinghaus (1965) and later by Fan et al. (2010). In support, early work by Fencl et al. (1969) reported leftward shifts in both cerebrovascular and ventilation responses to CO_2_ following a 5 day administration of sodium bicarbonate (inducing metabolic alkalosis), while chronic metabolic acidosis with ammonium chloride elicited an opposite effect on these CO_2_ responses. High altitude studies have reported a leftward shift in the ventilatory response to CO_2_ (Crawford and Severinghaus, 1978; Mathew et al., 1983; Somogyi et al., 2005; Ainslie and Burgess, 2008; Fan et al., 2010), which can be predicted by modeling the acid-base changes in the CSF compartment (Duffin, 2005). Using CO_2_ supplementation, Cruz et al. (1980) demonstrated that the leftward shift in the ventilatory response to CO_2_ observed following 75 h of altitude exposure can be abolished by preventing the respiratory alkalosis (and subsequent negative base excess). These findings suggest that the changes in acid-base balance are responsible for the resetting of resting CBF and the leftward shifts in both cerebrovascular and ventilatory responses to CO_2_ during acclimatization to high altitude. In the present study, the leftward shift in the cerebrovascular CO_2_ reactivity at ALT16 was abolished when the MCAv changes were plotted against [H^+^] instead of PETCO_2_ (Figure 4). As a result, the midpoints of the sigmoid curves appeared at similar [H^+^] (~37 nEq/L) across all three time-points (Figure 4B). From this, we speculate that the observed leftward shift of the sigmoid curve at ALT16 is likely a consequence of reduced [${\text{HCO}}_{3}^{-}$] in the CSF, resulting from the prolonged exposure to hypoxia-induced hypocapnia. Moreover, our findings indicate that the cerebral reserves for vasoconstriction and vasodilation are most optimal around 37 nEq/L H^+^ concentration, regardless of altitude or acclimatization status. Our data support the notion that H^+^-mediated cerebrovascular control is highly conserved.

### Altered cerebrovascular reserves at high altitude

The midpoint of the sigmoid cerebrovascular reactivity curve represents the optimization point between maximal vasoconstriction and vasodilation (Battisti-Charbonney et al., 2011). A departure of resting PaCO_2_ from the midpoint toward the lower plateau lowers the vascular reserve for further constriction, while increasing PaCO_2_ toward the upper plateau lowers the vascular reserve for dilation (Figure 4A). In addition, outside the linear portion of the sigmoid curve, the CBF responsiveness to changes in CO_2_ is blunted. Together these changes in PaCO_2_ and associated reductions in vascular reserve for constriction and dilation limit the blood vessels' ability to regulate CBF against perturbations in either PaCO_2_ or ABP. As reported in our previous study (Subudhi et al., 2014a), hyperventilation at 5260 m lowered resting PaCO_2_ by ~12 mmHg at ALT1 and by ~17 mmHg at ALT16. These reductions in resting PaCO_2_ lowered the vascular constriction reserve by ~30% upon ascent to 5260 m and following acclimatization, despite the compensatory leftward shift in the cerebrovascular CO_2_ reactivity curve at ALT16 (Figure 4A). Our present findings indicate that hyperventilation-induced hypocapnia reduced the vascular reserve for vasoconstriction at high altitude, thereby blunting the vessel's ability to respond to changes in CO_2_ (and presumably blood pressure) at 5260 m. Importantly, this reduced vascular reserve and associated blunting of the cerebral vessels' responsiveness could potentially account for the impaired cerebral autoregulation observed during acute and chronic exposure to hypoxia, as previously reported by Subudhi et al. (2014b) and others (Jansen et al., 2000; Ainslie et al., 2008; Iwasaki et al., 2011).

### Confounding influence of background hypoxia and blood pressure

The effect of high altitude exposure on cerebrovascular CO_2_ reactivity has been examined by a number of studies (Jensen et al., 1996; Jansen et al., 1999; Ainslie and Burgess, 2008; Fan et al., 2010, 2012, 2014; Lucas et al., 2011; Villien et al., 2013; Rupp et al., 2014; Flück et al., 2015; Willie et al., 2015). More recently, studies have assessed cerebrovascular CO_2_ reactivity in *background hypoxia* (Rupp et al., 2014; Flück et al., 2015; Willie et al., 2015), which has been shown to blunt the CBF response to CO_2_ (McPherson et al., 1987; Fan et al., 2013; Ogoh et al., 2014)—presumably by exhausting the dilatory response and reducing prostanoid synthesis (Leffler et al., 1986). Those studies have found cerebrovascular CO_2_ reactivity to be either reduced (Rupp et al., 2014; Flück et al., 2015), or unchanged (Willie et al., 2015) following ascent to high altitude, while ventilatory responsiveness to CO_2_ was enhanced. In contrast, studies assessing cerebrovascular CO_2_ reactivity in background hyperoxia have consistently found it to be elevated with high altitude ascent (Fan et al., 2010, 2012, 2014) and prolonged exposure to hypoxia (Poulin et al., 2002), coinciding with enhanced ventilatory CO_2_ sensitivity. Due to the confounding effects of hypoxia, the cerebrovascular responses to CO_2_ assessed in *background hypoxia* may not necessarily reflect true cerebrovascular CO_2_ reactivity *per se* and thus should be interpreted with caution.

In agreement with our previous report using linear fitting (Fan et al., 2014), we found cerebrovascular CO_2_ reactivity to be enhanced by 85% upon ascent at ALT1, while no further increased was observed following the acclimatization period in this study (Table 1). Similar to other high altitude studies (Flück et al., 2015; Willie et al., 2015), we observed large elevations in blood pressure during CO_2_ breathing (Fan et al., 2014). Since increases in systemic blood pressure influence the CBF response to CO_2_ (Betz, 1968) and thus confounds the measurement of cerebrovascular CO_2_ reactivity (Regan et al., 2014), we attribute this smaller additional increase in cerebrovascular CO_2_ reactivity at ALT16 to the constant ABP during the rebreathing trials analyzed in this study. Therefore, it should be acknowledged that the previously reported changes of cerebrovascular CO_2_ reactivity at high altitude reflect the CBF response to the combined effects of both CO_2_ *and* arterial blood pressure, rather than CO_2_ alone. However, we cannot exclude the possibility that the lack of increase at ALT16 compared to ALT1 may be due a lower subject number.

### Limitations

One of the critiques when using TCD-measured MCAv as an index of CBF is the assumption that middle cerebral artery (MCA) diameter does not change. Due to the exponential dependency of the vessel cross-sectional area on the diameter, small changes in MCA diameter would result in considerable errors in CBF estimates with TCD. As mentioned in our previous study (Fan et al., 2014), we assessed MCAv changes in a background of hyperoxia (PETO_2_ > 250 mmHg), thus excluding any confounding dilatory effects of hypoxia on MCA diameter in our measurements. While early magnetic resonance imaging (MRI) studies found no changes in MCA diameter during changes in PETCO_2_ between 24 and 45 mmHg (Valdueza et al., 1997; Serrador et al., 2000), more recent and higher resolution MRI studies reported changes in MCA diameter (1.2–8%) during voluntary hyperventilation (PETCO_2_: 27–24 mmHg) and hypercapnia (PETCO_2_: 45–51 mmHg) (Coverdale et al., 2014; Verbree et al., 2014). The major implications of these MCA diameter changes are *overestimation* of CBF at low PaCO_2_, and *underestimation* of CBF at high PaCO_2_ with TCD. In agreement, Willie et al. (2015) found TCD to underestimate cerebrovascular CO_2_ reactivity when compared to the vascular Doppler ultrasound method. However, we contend that our assessment of CBF with TCD estimates are reliable since: (i) Caputi et al. (2014) have shown similar values of cerebrovascular CO_2_ reactivity using MRI and TCD estimates of CBF response to 5% CO_2_ breathing; and (ii) all the temporal indices such as the midpoint and range of the sigmoid curve would be unaffected by any underestimation of CBF with TCD. Another important consideration when interpreting our findings is our use of arterial pH and acid-base buffering as a surrogate for CSF pH changes at high altitude. Dempsey et al. (1974) previously reported consistent pH differences (~0.08), and similar [${\text{HCO}}_{3}^{-}$] between arterial and lumbar spinal fluid in seven subjects at sea-level, and following 1, 8 h, and 3–4 weeks at 3100 m. Moreover, they found identical pH compensation (66%) in both arterial and lumbar spinal fluid compartments following acclimatization to high altitude. The data from Dempsey et al. (1974) thus support the use of arterial acid-base changes as an index of CSF changes during acute and chronic exposure to high altitude.

## Conclusion

The present study is the first to demonstrate a remodeling of the sigmoid cerebrovascular CO_2_ reactivity curve following acclimatization to high altitude. Our data indicates that the increase in cerebrovascular CO_2_ reactivity upon ascent to high altitude is mediated by enhanced H^+^ sensitivity of the cerebral vasculature. Meanwhile, acclimatization to 5260 m leads to a leftward shift in cerebrovascular CO_2_ reactivity which appears to be mediated by an altered acid-base buffering following prolonged exposure to hypoxia. We speculate that such cerebrovascular resetting is critical for maintaining normal cerebrovascular control and preventing cerebral hypoperfusion from the prolonged severe hypocapnia associated with high altitude acclimatization.

## Author contributions

JF, AS, BK, AL, and RR contributed to conception and design of the experiments. JF and AS performed the experiments. JF, AS, and JD anaylsed the data. JF, AS, JD, BK, and RR interpreted the results of the experiments. JF, AS, and JD prepared the figures and drafted the manuscript. JF, AS, JD, BK, AL, and RR edited and revised the manuscript. JF, AS, JD, BK, AL, and RR approved the final version of the manuscript.

## Funding

This study was supported by the Swiss National Science Foundation and the Faculty of Medicine of the University of Geneva. The overall AltitudeOmics study was funded, in part, by grants from the U.S. Department of Defense (W81XWH-11-2-0040 TATRC to RR, and W81XWH-10-2-0114 to AL); the Cardiopulmonary and Respiratory Physiology Laboratory, University of Oregon; the Altitude Research Center and the Charles S. Houston Endowed Professorship, Department of Emergency Medicine, School of Medicine, University of Colorado Denver.

### Conflict of interest statement

The authors declare that the research was conducted in the absence of any commercial or financial relationships that could be construed as a potential conflict of interest.
